# Errors and omissions in hospital prescriptions: a survey of prescription writing in a hospital

**DOI:** 10.1186/1472-6904-9-9

**Published:** 2009-05-13

**Authors:** Laura Calligaris, Angela Panzera, Luca Arnoldo, Carla Londero, Rosanna Quattrin, Maria G Troncon, Silvio Brusaferro

**Affiliations:** 1 Chair of Hygiene, Department of Experimental and Clinical Pathology and Medicine, University of Udine, 33100 Udine, Italy; 2 Risk Management Unit, University Hospital of Udine, 33100 Udine, Italy; 3 Pharmacy Unit, University Hospital of Udine, 33100 Udine, Italy

## Abstract

**Background:**

The frequency of drug prescription errors is high. Excluding errors in decision making, the remaining are mainly due to order ambiguity, non standard nomenclature and writing illegibility. The aim of this study is to analyse, as a part of a continuous quality improvement program, the quality of prescriptions writing for antibiotics, in an Italian University Hospital as a risk factor for prescription errors.

**Methods:**

The point prevalence survey, carried out in May 26–30 2008, involved 41 inpatient Units. Every parenteral or oral antibiotic prescription was analysed for legibility (generic or brand drug name, dose, frequency of administration) and completeness (generic or brand name, dose, frequency of administration, route of administration, date of prescription and signature of the prescriber). Eight doctors (residents in Hygiene and Preventive Medicine) and two pharmacists performed the survey by reviewing the clinical records of medical, surgical or intensive care section inpatients. The antibiotics drug category was chosen because its use is widespread in the setting considered.

**Results:**

Out of 756 inpatients included in the study, 408 antibiotic prescriptions were found in 298 patients (mean prescriptions per patient 1.4; SD ± 0.6). Overall 92.7% (38/41) of the Units had at least one patient with antibiotic prescription. Legibility was in compliance with 78.9% of generic or brand names, 69.4% of doses, 80.1% of frequency of administration, whereas completeness was fulfilled for 95.6% of generic or brand names, 76.7% of doses, 83.6% of frequency of administration, 87% of routes of administration, 43.9% of dates of prescription and 33.3% of physician's signature. Overall 23.9% of prescriptions were illegible and 29.9% of prescriptions were incomplete. Legibility and completeness are higher in unusual drugs prescriptions.

**Conclusion:**

The Intensive Care Section performed best as far as quality of prescription writing was concerned when compared with the Medical and Surgical Sections.

Nevertheless the overall illegibility and incompleteness (above 20%) are unacceptably high. Values need to be improved by enhancing the safety culture and in particular the awareness of the professionals on the consequences that a bad prescription writing can produce.

## Background

Adverse drug events (ADEs), usually defined as injuries caused by the use of a drug, are a major health concern for the patient in most clinical settings.[1]

It has been estimated that ADEs account for approximately 5% of all hospital admissions, occur during 10–20% of hospitalisations and are responsible for 7–9% of hospitalisation days.[2]

Some ADEs are caused by errors called medication errors that have similar consequences as well as lowering patient satisfaction.[3] Chart reviews of inpatients reveal that over half of all hospital medication errors occur at the interfaces of care.[4]

If we consider harm caused by any error, medication error is the fourth most frequent category among all sentinel events collected by the Joint Commission between January 1995 and June 2008 after wrong site surgery, suicide and op/post op complication.

A medication error can occur at any step of the medication use process: prescribing, transcribing, dispensing and administering. Prescribing and administering errors are the two most frequent types of medication errors, but while 48% of the former can be intercepted, only 2% of the latter are intercepted.[5]. The reported frequency of prescription errors varies between 39% [6] and 74% of all medication errors [7] in specific settings. Ridley states that almost 50% of all prescription errors in Intensive Care Units (ICUs) are due to four categories: not writing the order according to the formulary, ambiguous medication order, non standard nomenclature and writing illegibility.[8]

A broad definition of prescribing error includes errors in decision making and errors in prescription writing.[9] Prescribing errors involving decision making include a wrong choice for the patient (due to allergies, interactions between two drugs, presence of liver or renal failure, wrong molecule, dose or route of administration, etc.). Prescription errors in prescription writing, instead, involve illegibility, ambiguous abbreviations, lack of an important piece of information such as date of prescription, dose, route, frequency of administration, etc.[10] Since the latter can be more easily determined and detected through chart review, we focused our attention on them.

The aim of this study is to analyse, as a part of a continuous quality improvement program, the quality of prescription writing for antibiotics, in an Italian University Hospital. We did not analyse the appropriateness of the molecule choice, but we evaluated the completeness and legibility of information present in the clinical records as risk factors for prescription errors.

## Methods

The study, a point prevalence survey, took place between 26–30 May 2008 in a North-Eastern University hospital. All 41 inpatient Units were involved, except for ophthalmology and dermatology since their antibiotic use, when present, is mainly topical, an administration route not considered in this survey.

For study purpose Units were grouped together in medical, surgical and intensive care sections, as follows:

• Medical section: cardiology, haematology, infectious diseases, internal medicine, nephrology, neurology, oncology, pediatrics, post acute care, pulmonology, radiotherapy, rheumatology, pain control, nursery.

• Surgical section: general surgery, maxillofacial surgery, plastic surgery, vascular surgery, heart surgery, vertebral (spine) surgery, neurosurgery, urology, gastroenterology, orthopedics and traumatology, obstetrics, gynecology, otorhinolaryngology.

• Intensive Care section: anesthesia and intensive care unit, medical intensive care unit, neonatology, coronary unit.

The surveyors looked at the clinical record, both nursing and medical data, of the inpatients present in ward at 8.00 AM on the day of the survey. In the hospital different formats exist for recording drug prescriptions. A healthcare worker (HCW) was in charge of giving information to the surveyors in case the clinical record was not clear enough. All antibiotic prescriptions for parenteral or oral use were included in the survey. Every Unit was surveyed in one single day. Patient's age, gender and number of admitted patients were recorded. For those patients who had any parenteral or oral antibiotic prescriptions we collected further information: antimicrobial agent, dose, date of prescription, prescriber signature, frequency of administration, route of administration, indication for given therapy or target for prophylaxis in medical records and presence of microbiological culture before therapy. When the indication was surgical prophylaxis it was specified whether it was single dose or lasting ≤ 24 h or > 24 h.

Each prescription was analysed for: legibility (generic or trade name, dose, frequency of administration) and completeness (generic or brand name, dose, frequency of administration, route of administration, date of prescription and signature of the prescriber). Each item was classified as compliant when it was filled in and legible; if partially compliant it was classified as non compliant. The total completeness and legibility were calculated considering all the specific items.

The Anatomical Therapeutic Chemical (ATC) classification system was used to class the antibiotics.

The adopted definition of completeness was "having all necessary parts or components" while the one adopted for legibility stated "easily readable by someone who is not familiar with the context examined".

Eight doctors (residents in Hygiene and Preventive Medicine) and 2 pharmacists performed the survey. They gave each patient an identifying code, so that the data collected were anonymous.

A meeting was held before starting the survey so that the surveyors could analyse the items on the form and standardize data collection with the aim of reducing the inter operator variability. To this end pairs of surveyors were created to visit each ward, too.

This observational study was performed in agreement with the local ethical committee in compliance with the Italian law.

Data were processed using the software program SPSS version 12.0. The statistical analysis was performed using the Chi-Square, non parametric k-sample (Kruskal-Wallis) and 2-sample (Mann Whitney) tests assuming as significant a p value ≤ 0.05.

## Results

Out of 756 patients included in the study, 408 antibiotic prescriptions were found in 298 patients (mean prescriptions per patient 1.4; SD ± 0.6). Overall, 39.4% of patients used antibiotic and 92.7% (38/41) of the Units had at least one patient with antibiotic prescription. Table 1 shows the percentage of patients with antibiotic prescription classified by section.

**Table 1:** Distribution of patients and prescriptions in the three areas considered.

**Section ** **(n. units)**	**% Patients with antibiotic prescription ** **(patients with prescription/patient observed)**	**Antibiotic prescriptions ** **(n.)**	**n. antibiotics/patient ** **(Mean; Standard Deviation)**
Medical Section (16)	43,1 (155/360)	221	1,4 ± 0,7
Surgical Section (18)	34,0 (106/312)	130	1,2 ± 0,4
Intensive Care Section (7)	44,0 (37/84)	57	1,5 ± 0,6

**Total**	**39,4** **(298/756)**	**408**	**1,4 ± 0,6**

Antibiotic prescriptions from the medical section were significantly higher than prescriptions from the surgical section (p = 0.019), whereas the mean number of prescriptions per observed patient proved to be significantly higher in intensive care section (p = 0.008) and medical section (p = 0.04) compared to the surgical section.

For 165/408 (40.4%) prescriptions the indication was prophylaxis both medical and preoperatory; the remaining 243/408 (59.6%) had a therapeutic indication.

Of the 243 therapeutic prescriptions overall 55.6%(135/243) had a previous written request for a microbiological culture without differences among different areas.

Written reasons for prescribing antibiotic were found in 58.1%(237/408) of the prescriptions, more frequently for therapeutic indication 166/237 (70%) than for prophylaxis 71/237 (30%); in the remaining cases it was necessary to request information from the medical or nursing staff.

In medical section written reasons for using the antibiotic were present in 65.2%(144/221) of prescriptions, in surgical section in 46.2%(60/130) and in intensive care section in 57.9%(33/57) of the cases.

Prescriptions for perioperative prophylaxis in the surgical section were for more than 24 hours in 87.7% (79/90) of the cases.

To obtain a global view of legibility and completeness in the hospital antibiotic prescriptions we analysed drug name, dose and frequency and route of administration, prescription date and signature classified by section [see Additional file 1].

A more detailed analysis showed that the legibility versus illegibility of the drug's name was higher in the medical section than in the other two sections (p = 0.003). Intensive care completeness was significantly higher than the other two as far as dose (p < 0.001), frequency of administration (p = 0.035) and prescription date (p < 0.001) are concerned.

In the medical and intensive care sections the route of administration was completed in a significant greater portion of the prescriptions than in the surgical section (p < 0.001 and p < 0.035 respectively). Overall 23.9% of prescriptions were illegible and 29.9% of prescriptions were incomplete.

The distribution of legibility and completeness by antibiotic category is shown in additional file 2 [see Additional file 2].

## Discussion

The study highlights the need to pay attention to antibiotic prescription writing: in fact 1 in 4 prescriptions were not fully completed or were illegible. We think this is a field that could be improved, particularly for some items, like dosage legibility prescription, date and physician's signature.

We found a widespread use of antibiotics: almost all Units (92.7%) on the day of the survey had at least one patient with an antibiotic prescription.

This is not unusual in acute care hospitals as reported in other studies.[11,12] Such a wide use implies also that potential antibiotic medication errors are events to attentively control and prevent.

Our study focused attention on formal quality characteristics that is part of the medication error risk. Certainly this risk should be evaluated hospital by hospital and, eventually in each department or Unit, possibly adopting proactive methods like the Health Failure Mode Effect Analysis (HFMEA).

Nevertheless, we think that data presented in this paper can serve to increase health care workers' awareness of drug use safety, and specifically of written communication, i.e. how to fill in clinical documentation precisely.

Monitoring these aspects is quite simple since based on existing clinical documentation. We performed a hospital wide survey but this is not strictly necessary in a routine program since a few cases per Unit would be enough to show attitudes and behaviours of the professional teams.

If any hospital quality and safety oriented team would adopt the same methodology it should involve some professionals to read prescriptions: no specific training is required but the recommendation that professionals are different from those of the observed Units.

In fact doctors and nurses have to use this information to decide on further clinical actions: if they are not able to find or read and understand written prescriptions, they can immediately realise the risks related to patient safety.

Through this methods we can detect also the use of acronyms that we know are a risk factor when different professionals do not share their meaning, or worse, they are misinterpreted.[13].

Our data show that Intensive Care Units have some characteristics we should consider:

• patients with antibiotic prescriptions and mean number of prescriptions per observed patient are higher than in the other sections because of the expected severity and typology of inpatients;

• they show the best performance in terms of completeness in all items, except for the signature of the physician that remains unfulfilled. These data may mirror the fact that the Intensive Care Units are selected settings where the patient safety culture is more widespread because they often have to deal with critical situations like emergencies. The diffusion of risk reduction strategies such as protocols or checklists in these settings have long been appreciated and are more frequently used in these sections.

Written reasons for antibiotic prescriptions cover approximately half of the cases (58.1%), more frequently if the indication is therapeutic. Microbiological cultures are requested before starting the therapy in a percentage slightly over the half as well (55.6%). In our opinion both aspects should be improved and they must be part of a wider quality improvement program related to antibiotic use appropriateness.

We found that perioperatory prophylaxis lasting > 24 h was high (87.8%) in spite of the international guidelines recommending a short or ultra short first choice prophylaxis, leaving to a limited number of selected patients the possibility to extend it over one day [14]. This finding, however, reflects a widespread approach common to both Italian [15] and worldwide [16] hospital settings. We did not collect information on surgical patients case-mixes and thus it is not possible to judge the appropriateness of the extended perioperative prophylaxis. Further studies could address this issue and, in our case, evaluate the best strategies to support the adoption of existing guidelines in future [17,18].

Legibility and completeness are higher in unusual drugs prescriptions.

In general, there are consistent differences in legibility and completeness when different chemical principles are concerned but we cannot compare all these percentages, because, in the case of specific drugs (i.e. Antimycotics for systemic use), we analysed only one hospital and the performance can be influenced by a limited number of Units prescribing that chemical principle.

Further important but simple directions that come out as priorities from this survey are the necessity to print prescriptions and the need to record the route, dose and frequency of administration of the drug. The lack of prescription date and doctor's signature were the most critical areas in terms of prescription completeness, both were absent in more than 50% of the prescriptions.

We think that a systematic use of feedback together with the adoption of formats where spaces for prescription date, signature of the physician and route of administration are more emphasised would simplify the prescriber's task.

It is reported that computerized physician order entry and computerised physician decision support, in fact, significantly reduce prescription errors improving drug safety [19]. Nevertheless, an investment in information technology is not always feasible, at least not in the short term particularly in those healthcare settings with economic restraints. In these circumstances a few simple recommendations to lower the prescription error rate can be adopted, since they are useful, cheap and easy to apply: i.e. the introduction of an integrated patient therapy record, the standardization of the patient's record format throughout the hospital, a high completeness and legibility of the prescription, the discouraging of verbal prescriptions and, if any are given, the introduction of a read-back procedure and finally an active involvement and education of the patient in the current knowledge of his/her drug therapy [20].

This study analysed only the legibility and completeness as risk factors for prescription errors but despite this, we think it has an operative relevance and it is essential in improving quality of healthcare and reducing errors.

A limit of the study is that only some of the risk factors in prescription writing can be detected through chart review, thus for instance the potential error of omission is an aspect that cannot be detected through this methodology but could be considered for further studies that involve the direct observation of the professionals.

The paper addresses an important issue of antibiotic safety that reflects a global problem. In fact this is not only a problem in Italy but also in other hospitals around the world. It would be also interesting to include in a further study numerous hospitals. The magnitude of this problem could be even more severe in the less resourced countries such as Africa and Asia.

The findings confirm that there is a problem that needs to be attended to and serve to sensitise stakeholders in health delivery about this issue.

## Conclusion

The survey confirms the extensive use of antibiotics in an acute care hospital.

Written reason for the use of an antibiotic is poor as is the request for microbiological culture in case of therapeutic indication.

Overall legibility is good in more than three out of four cases, while completeness is poor mainly concerning the date of prescription and the signature of the physician.

The feedback of the objective data to the Units is a great opportunity to improve the awareness of safety and to stress the need for accuracy in prescription writing. As the measurements are objective they can be repeated to monitor trends over time.

Since several easily identified risk factors are associated with a large proportion of medication prescribing risk factors, an intervention is needed to enhance the safety culture in all settings by improving clinical documentation and through enhanced the professional awareness of potential medication errors related to bad prescription writing.

## Competing interests

The authors declare that they have no competing interests.

## Authors' contributions

All authors have substantially contributed to the research. Specifically CL, BS conceived the idea for the study, and PA, AL, LC, TMG, participated in its design. All authors were involved in data collection and CL, PA, AL performed the analysis and interpretation of data and the statistical analysis. CL, PA, AL, LC, QR have been involved in drafting the manuscript and BS and TMG critically revised it. All authors read and approved the final manuscript.

## Pre-publication history

The pre-publication history for this paper can be accessed here:



## Supplementary Material

Additional file 1 **Legibility and completeness of antibiotic prescription by section (n. 408 prescriptions)**. the data provided represent the legibility and completeness of antibiotic prescription in the medical section, surgical section and intensive care section. Click here for file

Additional file 2 **Distribution of legibility and completeness of prescribed drugs for antibiotic category (n. 408 prescriptions)**. the data provided represent the legibility and completeness of antibiotic prescription related to antibiotic type. Click here for file

## References

[B1] Leape LL, Kabcenell A, Berwick DM (1998). Reducing adverse drug events.

[B2] Hardmeier B, Braunschweig S, Cavallaro M (2004). Adverse drug events caused by medication errors in medical inpatients. Swiss Med Wkly.

[B3] Morimoto T, Gandhi TK, Seger AC, Hsieh TC, Bates DW (2004). Adverse drug events and medication errors: detection and classification methods. Qual Saf Health Care.

[B4] Rozich JD, Resar RK (2001). Medication Safety: One organization's approach to the challenge. JCOM.

[B5] Bates DW, Cullen DJ, Laird N, Petersen LA, Small SD, Servi D, Laffel G, Sweitzer BJ, Shea BF, Hallisey R (1995). Incidence of adverse drug events and potential adverse drug events: Implications for prevention. JAMA.

[B6] Leape LL, Brennan TA, Laird N, Lawthers AG, Localio AR, Barnes BA, Hebert L, Newhouse JP, Weiler PC, Hiatt H (1991). The nature of adverse events in hospitalized patients. Results of the Harvard Medical Study II. N Engl J Med.

[B7] Fortescue EB, Kaushal R, Landrigan CP, McKenna KJ, Clapp MD, Federico F, Goldmann DA, Bates DW (2003). Prioritizing strategies for preventing medication errors and adverse drug events in pediatric inpatients. Pediatrics.

[B8] Ridley SA, Booth SA, Thomson CM, the Intensive Care Society's Working Group on Adverse Incidents (2004). Prescription errors in UK critical care units.

[B9] Dean B, Barber N, Schachter M (2000). What is a prescribing error?. Qual Health Care.

[B10] Lesar TS, Bryceland L, Stein DS (1997). Factors related to errors in medication prescribing. JAMA.

[B11] Borg MA, Zarb P, Ferech M, ARMed Project Group (2008). Antibiotic consumption in southern and eastern Mediterranean hospitals: results from the ARMed project. J Antimicrob Chemother.

[B12] Davey P, Brown E, Fenelon L, Finch R, Gould I, Hartman G, Holmes A, Ramsay C, Taylor E, Wilcox M, Wiffen P (2005). Interventions to improve antibiotic prescribing practices for hospital inpatients. Cochrane Database of Systematic Reviews.

[B13] American Hospital Association; American Society of Health-System Pharmacists; Hospitals & Health Networks (2005). Medication safety issue brief. Eliminating dangerous abbreviations, acronyms and symbols. Hosp Health Netw.

[B14] Mangram AJ, Horan TC, Pearson ML, Silver LC, Jarvis WR, The Hospital Infection Control Practices Advisory Committee (1999). Guideline for prevention of surgical site infection. Infect Control Hosp Epidemiol.

[B15] Dinelli F, Catalani V, Porretta A, Costa AL, Francone C, Privitera G (2006). Audit sulla modalità di effettuazione della profilassi antibiotica perioperatoria in una Azienda Ospedaliero-Universitaria. GIIO.

[B16] Bratzler DW, Houck PM, Richards C, Steele L, Dellinger PE, Fry DE, Wright C, Ma A, Carr K, Red L (2005). Use of antimicrobial prophylaxis for major surgery: baseline results from the national surgical infection prevention project. Arch Surg.

[B17] Istituto Superiore di Sanità, CeVEAS, Piano Nazionale Linee Guida (2006). Antibioticoprofilassi perioperatoria nell'adulto.

[B18] Regione Autonoma Friuli Venezia Giulia (2000). Linee guida per la profilassi antibiotica in chirurgia.

[B19] Bates DW, Cohen M, Leape LL, Overhage MJ, Shabot MM, Sheridan T (2001). Reducing the frequency of errors in medicine using information technology. J Am Med Inform Assoc.

[B20] Weingart SN, Toth M, Eneman J, Aronson MD, Sands DZ, Ship AN, Davis RB, Phillips RS (2004). Lessons from a patient partnership intervention to prevent adverse drug events. Int J Qual Health Care.

