# Supplementary material for: Errors and omissions in hospital prescriptions: a survey of prescription writing in a hospital
Source: BMC Clin Pharmacol. 2009 May 13;9:9. doi: 10.1186/1472-6904-9-9 (PMC2695418; doi:10.1186/1472-6904-9-9)
Supplement: Additional file 1 — Legibility and completeness of antibiotic prescription by section (n. 408 prescriptions). the data provided represent the legibility and completeness of antibiotic prescription in the medical section, surgical section and intensive care section. [file 1472-6904-9-9-S1.doc]

**Additional file 1**

Legibility and completeness of antibiotic prescription by section (n. 408 prescriptions).

| **Section:**  **n. prescriptions** | **Legibility** * | | | | | | | **Completeness** ^†^ | | | | | | | | | | | | |
| --- | --- | --- | --- | --- | --- | --- | --- | --- | --- | --- | --- | --- | --- | --- | --- | --- | --- | --- | --- | --- |
|  | **Drug** ^‡^ | | **Dose** | | **Frequency** ^§^ | | **Total°** | **Drug** ^‡^ | | **Dose** | | **Frequency** ^§^ | | **Route** \|\| | | **Date** ^¶^ | | **Signature**** | | **Total°** |
|  | **%** | **n.** | **%** | **n.** | **%** | **n.** | **%** | **%** | **n.** | **%** | **n.** | **%** | **n.** | **%** | **n.** | **%** | **n.** | **%** | **n.** | **%** |
| **Medical Section (n.221)** | **83,7** | 185 | **67,9** | 150 | **77,4** | 171 | **76,3** | **96,4** | 213 | **71,5** | 158 | **78,3** | 173 | **93,7** | 207 | **39,4** | 87 | **31,2** | 69 | **68,4** |
| **Surgical Section**  **(n.130)** | **70** | 91 | **66,9** | 87 | **78,5** | 102 | **71,8** | **95,4** | 124 | **77,7** | 101 | **85,4** | 111 | **74,6** | 97 | **42,3** | 55 | **40** | 52 | **69,2** |
| **Intensive Care Section (n.57)** | **80,7** | 46 | **80,7** | 46 | **94,7** | 54 | **85,4** | **93** | 53 | **94,7** | 54 | **100** | 57 | **98,5** | 51 | **64,9** | 37 | **26,3** | 15 | **79,6** |
| **Total** | **78,9** | 322 | **69,4** | 283 | **80,1** | 327 | **76,1** | **95,6** | 390 | **76,7** | 313 | **83,6** | 341 | **87** | 355 | **43,9** | 179 | **33,3** | 136 | **70,1** |

Legenda:

* Legibility: “easily readable by someone who is not familiar with the context examined”

† Completeness: “having all necessary parts or components”

‡ Generic or brand name

§ Number of doses per day

° Sum of the previous columns

|| Route of administration

¶ Date of prescription

** Signature of prescriber
