# Supplementary material for: Errors and omissions in hospital prescriptions: a survey of prescription writing in a hospital
Source: BMC Clin Pharmacol. 2009 May 13;9:9. doi: 10.1186/1472-6904-9-9 (PMC2695418; doi:10.1186/1472-6904-9-9)
Supplement: Additional file 2 — Distribution of legibility and completeness of prescribed drugs for antibiotic category (n. 408 prescriptions). the data provided represent the legibility and completeness of antibiotic prescription related to antibiotic type. [file 1472-6904-9-9-S2.doc]

## Additional file 2

**Distribution of legibility and completeness of prescribed drugs for antibiotic category (n. 408 prescriptions).**

| **Antibiotic category** | **Tot** * | **Legibility** ^†^ | | | | | | | **Completeness ^c^** | | | | | | | | | | | | |
| --- | --- | --- | --- | --- | --- | --- | --- | --- | --- | --- | --- | --- | --- | --- | --- | --- | --- | --- | --- | --- | --- |
|  |  | **Drug** ^§^ | | **Dose** | | **Frequency** \|\| | | **Total** | **Drug** ^§^ | | **Dose** | | **Frequency**\|\| | | **Route** ^¶^ | | **Date**** | | **Signature**^††^ | | **Total** |
|  | n. | **%** | n. | **%** | n. | **%** | n. | **%** | **%** | n. | **%** | n. | **%** | n. | **%** | n. | **%** | n. | **%** | n. | **%** |
| **Penicillins** | **136** | **75,7** | 103 | **68,4** | 93 | **85,3** | 116 | **76,5** | **94,1** | 128 | **80,1** | 109 | **86** | 117 | **87,5** | 119 | **46,3** | 63 | **28,7** | 39 | **70,5** |
| **Quinolone antibacterials** | **93** | **80,6** | 75 | **61,3** | 57 | **73,1** | 68 | **71,7** | **95,7** | 89 | **67,7** | 63 | **80,6** | 75 | **84,9** | 79 | **30,1** | 28 | **22,6** | 21 | **63,6** |
| **Cephalosporins and other beta-lactam antibacterials** | **67** | **82,1** | 55 | **85,1** | 57 | **73,1** | 53 | **80,1** | **100** | 67 | **89,6** | 60 | **77,6** | 52 | **83,6** | 56 | **31,3** | 21 | **22,4** | 15 | **67,4** |
| **Antimycotics for sistemic use** ^‡‡^ | **51** | **80,4** | 41 | **78,4** | 40 | **82,4** | 42 | **77,7** | **94,1** | 48 | **80,4** | 41 | **90,2** | 46 | **92,2** | 47 | **66,7** | 34 | **62,7** | 32 | **78,8** |
| **Other antibacterials** ^§§^ | **30** | **76,7** | 23 | **63,3** | 19 | **67** | 20 | **69** | **93,3** | 28 | **73,0** | 22 | **77** | 23 | **90** | 27 | **57** | 17 | **46,7** | 14 | **72,8** |
| **Macrolides and Lincosamides** | **11** | **72,7** | 8 | **81,8** | 9 | **100** | 11 | **84,8** | **100** | 11 | **72,7** | 8 | **90,9** | 10 | **100** | 11 | **45,5** | 5 | **36,4** | 4 | **74,3** |
| **Sulfonamides and Trimethroprim** | **9** | **100** | 9 | **11,1** | 1 | **100** | 9 | **70,4** | **100** | 9 | **11,1** | 1 | **100** | 9 | **100** | 9 | **77,8** | 7 | **66,7** | 6 | **75,9** |
| **TB Drugs** | **6** | **83,3** | 5 | **66,7** | 4 | **83** | 5 | **77,7** | **100** | 6 | **83** | 5 | **100** | 6 | **67** | 4 | **17** | 1 | **33,3** | 2 | **66,7** |
| **Tetracyclines** | **5** | **60** | 3 | **80** | 3 | **60** | 3 | **66,7** | **80** | 4 | **80** | 4 | **60** | 3 | **60** | 3 | **60** | 3 | **60** | 3 | **66,7** |

Legenda:

* Total prescriptions

† Legibility: “easily readable by someone who is not familiar with the context examined”

‡ Completeness: “having all necessary parts or components”

§ Generic or brand name

|| Number of doses per day

¶ Route of administration

**Date of prescription

†† Signature of prescriber

‡‡ Imidazole derivatives, triazole derivatives, other anitmycotics for sistemic use

§§ Glycopeptide antibacterials, Polymyxins, and other antibacterials
